# Pre-Exposure Prophylaxis for HIV Infection as a Public Health Tool

**DOI:** 10.1017/jme.2022.31

**Published:** 2022

**Authors:** Chris Beyrer, Sheena McCormack, Andrew Grulich

**Keywords:** HIV, Pre-Exposure Prophylaxis, Incidence, Public Health Tool

## Abstract

The efficacy of pre-exposure prophylaxis, PrEP, with antiviral agents for prevention of HIV infection has been demonstrated in multiple randomized controlled trials and demonstration projects. These trials have studied prevention at the individual level. The effectiveness of PrEP as a public health intervention to reduce HIV incidence at community and population levels is being actively evaluated but is less well described. In reviewing the available data on PrEP as a public health intervention, three significant examples have demonstrated success, and all have been among communities of gay, bisexual and other men who have sex with men (MSM).

## Introduction

The first use of antiviral drugs to prevent HIV transmission and acquisition was in the perinatal arena, initially with the first of the treatment drugs, Azidothymidine (AZT), and subsequently with Nevirapine.^1^ The first demonstration of the use of antiviral drugs for prevention of sexual acquisition, between men who have sex with men (MSM) and transgender women (TGW) who have sex with men, was the landmark iPrEx trial, in 2010, which showed a 41% reduction in HIV incidence among uninfected participants at risk for HIV infection with the use of daily oral Truvada (tenofovir disoproxil fumarate and emtricitabine).^2^ With optimal usage, oral PrEP has subsequently been found to be much more highly efficacious, over 99% in preventing sexual transmission between men, and 74 to 84% effective for people who inject drugs (PWID).^3^ The PWID data were from an individually randomized trial with daily oral Tenofovir alone among Thai PWID people in both men and women, though this trial did not differentiate between sex assigned at birth and current gender identity.^4^

Trials among cis-gender women at risk for HIV infection in Sub-Saharan Africa were disappointing, largely due to significantly lower rates of PrEP adherence.^5^ Discordant couples studies in Sub-Saharan African heterosexual couples did demonstrate good protection against HIV infection among uninfected partners, both male and female, in dyads where one partner was living with HIV infection.^6^ These trials, like iPrEx, evaluated efficacy with daily oral Truvada, but at the couples level and in generalized epidemic contexts, as opposed to HIV epidemics concentrated in particular populations, such as MSM in the UK, Australia, or the U.S. Later work led by French investigators demonstrated equivalent levels of protection with Truvada among cis-gender MSM using a coitally dependent or on-demand strategy, with 2:1:1 dosing regimens, again in concentrated epidemics among MSM in France and Canada.^7^

Two more recent trials of a novel long-acting injectable PrEP agent, cabotegravir given in bimonthly injections, show superiority against daily oral Truvada in both MSM and transwomen, and among cis-gender women in Sub-Saharan Africa.^8^ CAB-LA as prevention has not been implemented at this writing but will clearly be an important new prevention tool for many individuals and populations at risk.

Taken together, these trials established the efficacy of PrEP with Truvada at individual and couples levels. What is the evidence for effectiveness of PrEP as a public health strategy at wider network, community and population levels? And what is the evidence for this intervention in social and sexual networks of MSM, which have suffered some of the highest HIV inicidence rates in the global HIV pandemic?^9^ To answer this question, we must look to settings in which PrEP has been taken to scale and has reached enough members of social and sexual networks at risk to reduce not only individual level acquisition risks, but to impact community or population level incidence densities. It should be made clear that to date, what evidence is available for the effectiveness of PrEP on community level incidence has only been reported from concentrated epidemic contexts, not generalized ones, such as the high burden generalized HIV epidemics in Southern and Eastern Africa.

What is the evidence for effectiveness of PrEP as a public health strategy at wider network, community and population levels? And what is the evidence for this intervention in social and sexual networks of MSM, which have suffered some of the highest HIV inicidence rates in the global HIV pandemic? To answer this question, we must look to settings in which PrEP has been taken to scale and has reached enough members of social and sexual networks at risk to reduce not only individual level acquisition risks, but to impact community or population level incidence densities.

Before turning to these questions, however, it is vital to address the other interventions with evidence for efficacy in reducing transmission of HIV infection, including condom use, other behavior change interventions, and HIV treatment as prevention. While there has never been a formal randomized trial of condom use to prevent HIV infection, there is abundant evidence of the effectiveness of consistent condom use at the individual, couple and community level in limiting HIV spread. That said, concerns about behavioral disinhibition and reductions in condom use among those at risk have hampered PrEP programs in some settings and for some providers.^10^ Treatment as prevention has shown good evidence for protection of uninfected primary partners, beginning with HPTN 052, the seminal trial which demonstrated the impact of earlier initiation of antiretroviral therapy for index partners in discordant heterosexual and male same sex partnerships, data that was supported by subsequent larger cohort studies of discordant heterosexual and male same sex couples reporting inconsistent (or no) condom use.^11^

However, a series of large efficacy trials of treatment as prevention to reduce HIV incidence at community or population levels, including the ANRS trial in South Africa, the SEARCH study in Kenya and Uganda, and the Pop-ART trial in South Africa and Zambia all failed to demonstrate efficacy, suggesting that treatment alone may be insufficient to reduce community incidence densities in these generalized epidemic contexts.^12^ The efficacy of PrEP as a public health tool has to be understood as a function of both the effectiveness of PrEP in reducing the pool of susceptible persons, and the coverage of treatment and levels of viral suppression among persons living with HIV in the population. In the settings where there is evidence of the effectiveness of PrEP, treatment coverage has also been high, arguing for the synergistic effects of prevention and treatment with antiretroviral agents in lowering overall transmission risks and reducing incidence. Health system factors, including the presence or absence of national programs for health coverage, such as those in Australia, much of Western Europe, and the UK, are also critically important.

## Case Studies in PrEP for HIV Prevention

Among the first studies to demonstrate a population-level impact of PrEP was the Expanded PrEP Implemention of Communities New South Wales (EPIC-NSW) trial in that Australian State.^13^ Prior to the program, the HIV epidemic in NSW was highly concentrated among gay, bisexual and other MSM and incidence had remained stable for the decade leading up to 2015. Australia has long had a national health insurance scheme. EPIC-NSW was targeted to high risk HIV uninfected MSM in a rapid rollout from March 2016 to April 2018. The study team measured both HIV incidence in the study population of men on PrEP, and new HIV diagnoses among MSM in the State over the 12-month period before and 3 years after PrEP implementation. Among some 9,448 MSM enrolled and followed for up 3 years, 30 new infections were observed, all among non-adherent men, yielding a very low incidence in the cohort of 0.16/100 person years with robust confidence intervals.^14^ This was 92% lower than the historically-expected incidence of at least 2 per 100 person-years. There were some 285 new HIV diagnoses among MSM in NSW in 2015 in the 12 months before the PrEP rollout, and by 2019 there were 215, a decline of 25% (95% CI, 10.5-37.4). There was a 40% decline in recent infections, likely reflecting incidence trends.^15^ The study team concluded that rapid and targeted rollout of PrEP in this population of MSM led to significant declines in new infections at population levels.

The Australian health care system and its financing for PrEP also likely played a role in the success of the rollout. During the EPIC-NSW study, PrEP drug costs and all required laboratory testing was free to participants. Clinic fees were either free (for the large majority) or had a modest co-pay for private providers. There was a completely free option and most participants chose this plan. After the study, participants now receive PrEP through the nationally subsidized medication scheme. They pay $AUD40 (about USD $29) a month for generic TDF/FTC; or $A6.50 if they are in receipt of government benefits. Lab tests remain free and quarterly clinic visits are either free (at publicly funded sexual health clinics) or they pay a small co-pay (for general practitioners).

In England, UK, HIV incidence rates remained high in gay, bisexual and other MSM despite relatively high ART treatment coverage rates among MSM living with HIV infection.^16^ While PrEP efficacy in RCTs had been determined, its real world effectiveness in high risk communities was less well understood, and the concerns around behavioral disinhibition in high risk MSM suggested that effectiveness might be less than what had been seen in the trials. The PROUD study sought to investigate the real world use of PrEP among MSM in England who had reported unprotected anal sex in the recent past. The study design was an open label offer of immediate versus deferred PrEP for MSM meeting PrEP risk criteria. Some 544 MSM were enrolled, 275 in the immediate arm, and 269 in the deferred group. The incidence was strikingly high in the men offered deferred PrEP, at 9/100 person years (pyrs), and markedly lower in the men offered immediate PrEP, at 1.2/100 pyrs, leading to early cessation of the study as designed, and an offer of immediate PrEP to all participants. Importantly, sexually transmitted infections, common in both groups, did not significantly differ.

Although cost-effective, the funds required for a national program could not be determined as the demand for PrEP was unknown and the patented drug costly.^17^ Scale up was therefore implemented in the PrEP Impact trial using generic drug (TDF/FTC) with efforts to enrol from a broader population including transgender persons and heterosexual men and women with moderate success (approximately 1 in 20 from non-MSM populations). Half of the MSM offered PrEP enrolled, resulting in a 10% coverage overall, with no difference by ethnic group but lower amongst those aged 16-24 in whom only 6% took up PrEP.^18^ HIV infections amongst the 17,770 MSM enrolled through one of 157 sexual health clinics with 1 or more visits post-enrolment were compared to non-PrEP using MSM attending the clinics during the recruitment period who met the eligibility criteria for PrEP based on national surveillance data. There were 25 new infections amongst the PrEP Impact participants, only 1 of which was a possible biological failure.^19^ HIV incidence was markedly reduced at 0.13 per 100 pyrs (95% CI: 0.08-0.19) compared to 1.01 per 100 pyrs (95% CI: 0.93-1.09) amongst the non-PrEP users. Other STI were more common amongst PrEP users compared to the non-PrEP control attendees, but just over half of the PrEP users had no STI and a quarter accounted for 80% of the diagnoses, demonstrating that the burden of STIs is borne by a sub-population of PrEP users. Characteristics of those diagnosed with an STI were young age, born outside the UK, and Black Caribbean ethnicity, but the strongest predictor of an STI in follow-up was having had an STI in the year prior to enrolment. PrEP became available, free of charge with broad eligibility criteria in March 2020 as part of the UK’s National Health Service. Clinical care and required laboratory testing have been free of charge for STIs since 1917, and for HIV regardless of citizenship since 2012, in all UK nations.

In the US, which like the UK and Australia has a concentrated HIV epidemic among MSM, the most recent national surveillance data are from 2019, reported in 2021.^20^ These data demonstrate that PrEP uptake and use continues to show wide racial and ethnic disparities among US MSM, with some 63% of white MSM with a PrEP indication on the medication, only 14% of Latinx MSM, and fewer than 9% of Black MSM.^21^ While these data are troubling, and suggest the current US HIV prevention efforts are failing men of color, they also suggest that PrEP is reaching levels of coverage in some groups of MSM sufficient to impact incidence in those groups. Indeed, the CDC estimates that HIV incidence declined some 32% among White MSM from 2018 to 2019 nationally, a significant decline.^22^ Latino MSM incidence also declined, but only modestly. Overall, new diagnoses in MSM in the US have declined only some 8% from 2015 to 2019, indicating that the widening health disparities among US MSM continue to hinder prevention gains at national levels. Nevertheless, the finding that more than two thirds of men in a subgroup (Non-Hispanic White MSM) with an indication were on PrEP in 2019, and incidence in that population fell by a third, is highly encouraging. If the U.S. could overcome the social, structural and health systems barriers to PrEP faced by racial and ethnic minority MSM, significant HIV prevention benefits could be achieved.^23^

That the US, unlike the UK and Australia, does not have a national health system and does not assure access to health care for all its citizens, plays a significant role in the persistence of these health disparities. Indeed, the context of research studies, such as the HPTN 073 study of PrEP uptake and adherence among Black MSM, removal of the cost barriers and insurance coverage issues demonstrated both good uptake of PrEP among these men, good adherence at 12 months, and significant declines in HIV incidence among PrEP users compared to men who did not initiate PrEP.^24^

The consistent findings from New South Wales, the UK, and among White MSM in the US that PrEP can be an effective tool to reduce HIV incidence at population levels in concentrated epidemics among MSM is highly encouraging. The data on individual and couples level prevention among gay and bisexual men was already compelling, and indeed oral PrEP effectiveness has been higher among MSM than for other populations. This is an oral drug for a rectal exposure. The HIV epidemics among networks, communities, and populations of MSM have been stubbornly persistent in many settings.^25^ PrEP taken to scale can clearly change that reality. But achieving these gains requires rapid, equitable and widespread distribution, and marginalized men and their communities must be better served. New, low threshold models of PrEP access, distribution, and financing will likely be required to achieve epidemic control of HIV in many settings, including the U.S. where health care access limitations continue to burden communities and maintain the risk environment for too many.

## Note

This work was supported, in part, by a grant to Johns Hopkins University from Arnold Ventures. SMc was supported by the Medical Research Council (MC_UU_00004/03). AG was supported by a National Health and Medical Research Council Investigator Grant, 1173931. CB reports being an PI for an NIH R01 in Thailand. Gilead provided Truvada for this study.
